# Bone Microstructure and Regional Distribution of Osteoblast and Osteoclast Activity in the Osteonecrotic Femoral Head

**DOI:** 10.1371/journal.pone.0096361

**Published:** 2014-05-06

**Authors:** Cheng Wang, Xin Wang, Xiao-long Xu, Xue-ling Yuan, Wen-long Gou, Ai-yuan Wang, Quan-yi Guo, Jiang Peng, Shi-bi Lu

**Affiliations:** Institute of Orthopedics, Chinese PLA General Hospital, Haidian District, Beijing, P.R. China; Georgia Regents University, United States of America

## Abstract

**Objective:**

To detect and compare the bone microstructure and osteoblast and osteoclast activity in different regions of human osteonecrotic femoral heads.

**Methods:**

Osteonecrotic femoral heads were obtained from 10 patients (6 males, 4 females; Ficat IV) undergoing total hip arthroplasty between 2011 and 2013. The samples were divided into subchondral bone, necrotic, sclerotic, and healthy regions based on micro-computed tomography (CT) images. The bone microstructure, micromechanics, and osteoblast and osteoclast activity were assessed using micro-CT, pathology, immunohistochemistry, nanoindentation, reverse transcription polymerase chain reaction (RT-PCR), tartrate-resistant acid phosphatase staining and Western blotting.

**Results:**

(1) The spatial structure of the bone trabeculae differed markedly in the various regions of the osteonecrotic femoral heads. (2) The elastic modulus and hardness of the bone trabeculae in the healthy and necrotic regions did not differ significantly (*P* >0.05). (3) The subchondral bone and necrotic region were positive on TRAP staining, while the other regions were negative. (4) On immunohistochemical staining, RANK and RANKL staining intensities were increased significantly in the subchondral bone and necrotic region compared with the healthy region, while RUNX2 and BMP2 staining intensities were increased significantly in the sclerotic region compared with the necrotic region. (5) OPG, RANK, RANKL, RUNX2, BMP2, and BMP7 protein levels were greater in the necrotic and sclerotic region than in subchondral bone and the healthy region.

**Conclusion:**

The micromechanical properties of bone trabeculae in the necrotic region did not differ significantly from the healthy region. During the progress of osteonecrosis, the bone structure changed markedly. Osteoclast activity increased in subchondral bone and the necrotic region while osteoblast activity increased in the sclerotic region. We speculate that the altered osteoblast and osteoclast activity leads to a reduction in macroscopic mechanical strength.

## Introduction

Osteonecrosis of the femoral head is a common, refractory disease in orthopedics departments. Non-traumatic femoral head necrosis, which occurs frequently in young and middle-aged patients (30 to 50 years old), progresses rapidly and has a high disability rate. Many etiologies disrupt the blood circulation to the femoral head, causing different degrees of cell death within the femoral head, affecting osteocytes, bone marrow, and hematopoietic cells. The resulting necrosis gradually decreases the macroscopic mechanical strength in the necrotic region, which leads to collapse of the femoral head and ultimately osteoarthritis of the hip[1], [2]. However, the mechanism of osteonecrosis of the femoral head is unclear. Many studies have shown that the osteonecrosis repair process requires precisely coordinated bone resorption and bone formation. Osteoblasts promote bone formation while osteoclasts give rise to bone resorption, and each regulates the other. Osteoclasts have positive and negative regulatory effects on osteoblast function [3], and the formation, differentiation, and maturation of osteoclasts are regulated by various solubility factors released by osteoblasts [4]. However, this balance is disrupted under pathological conditions, causing abnormal bone structure and function, resulting in various bone diseases, such as osteonecrosis of the femoral head.

The relationship between the decrease in mechanical strength of the femoral head and the possible restoration of the femoral head is uncertain. Therefore, it is necessary to investigate the structure of bone trabeculae and the change in osteoblast and osteoclast activity in different regions of the femoral head to explore the process of necrosis and the mechanism of femoral head collapse.

The nanoindentation technique is a new method for measuring the properties of bone [5]. It can accurately measure the elastic modulus and hardness of fine bone structures, such as trabeculae and lamellar bone, and can be used to test the toughness of bone microstructures in fracture testing.

Using pathological and immunohistochemical staining, tartrate-resistant acid phosphatase (TRAP) staining, quantitative real-time reverse transcription-polymerase chain reaction (qRT-PCR), micro-computed tomography (micro-CT), and Western blotting, we assessed the change in bone microstructure, micromechanical strength of bone trabeculae, and activation of osteoblasts and osteoclasts in different areas of femoral head specimens that had undergone osteonecrosis. We hypothesized that there would be significant differences in the bone microstructure in different areas of these femoral heads. The viability of osteoclasts in the subchondral bone and necrotic region was enhanced, leading to bone absorption. The viability of osteoblasts in the sclerotic regions was also enhanced. In the process of femoral head osteonecrosis, the activity of osteoblasts and osteoclasts changed, leading to a reduction in macromechanical strength. Continuous mechanical load would lead to the evetual collapse of the femoral head.

## Materials and Methods

### Materials

Ten femoral heads (Ficat IV) were obtained from patients with non-traumatic femoral head osteonecrosis who underwent total hip replacement in our hospital from 2011 to 2013.The study was approved by the Ethics Committee of the General Hospital of the Chinese Peoples Liberation Army, and written informed consent was obtained from all of the participants.

### Micro-CT evaluation, three-dimensional (3D) reconstruction and bone histomorphometry analysis

**Figure 1:**
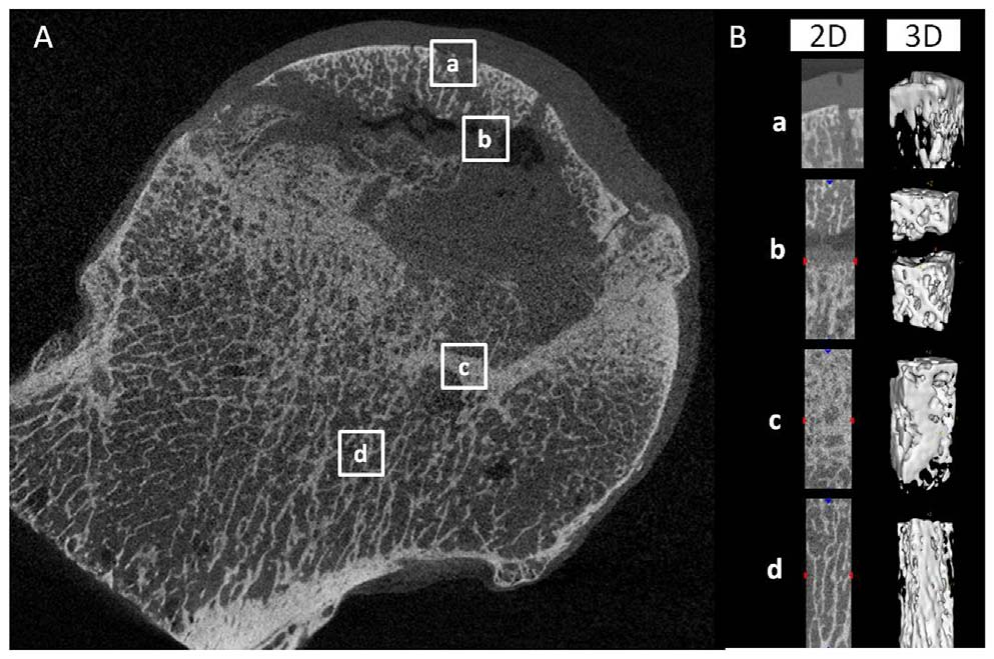
Two-dimensional slices and three-dimensional reconstruction of micro-CT images of the femoral heads. (A) Two-dimensional slices of micro-CT images of the subchondral bone (a), necrotic (b), sclerotic (c), and healthy(d) regions were distinguished according to bone mineral density. (B) The two-dimensional and three-dimensional reconstructed image of the white box regions.

Micro-CT (GE Explore Locus, USA) was performed on all femoral head samples with 45-µm resolution. The scanning protocol was 80 kV and 450 mA, with an isotopic resolution of 45 × 45 × 45 mm voxel size and an integration time of 14 ms. From the micro-CT images, the femoral heads were subdivided into subchondral bone, necrotic, sclerotic, and healthy regions (Figure 1). A volume of interest (VOI) was selected from these regions for three-dimensional reconstruction and the following bone parameters were analyzed: bone volume fraction (BV/TV%), trabecular number (Tb.N, mm), trabecular separation (Tb.Sp,µm), and trabecular thickness (Tb.Th,µm).All analyses were calculated as reported previously [6].

### Specimen preparation

**Figure 2:**
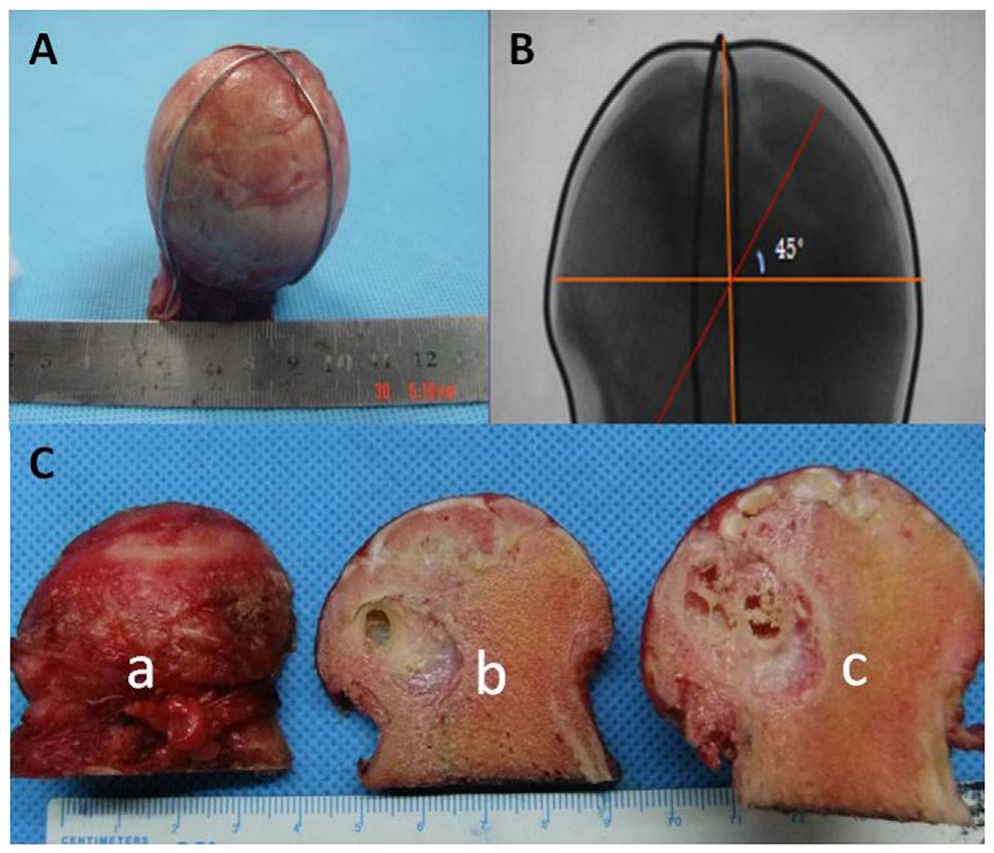
Cutting method of the femoral heads. The femoral heads were marked with an iron wire in the coronal plane and subjected to X-ray to confirm that all of the samples showed similar processes (A, B). The samples were divided into three parts: *a* for paraffin sections, *b* for real-time PCR and Western blotting, and *c* for undecalcified tissue sectioning and nanoindentation (C, D).

After the micro-CT scan, the fresh femoral head specimens were processed (Figure 2). Parts *a* and *c* were fixed in 4% paraformaldehyde for 2 weeks, and then processed to examine the pathology. Part *b* was cut into four regions (subchondral bone, necrotic, sclerotic, and healthy regions), and stored at −80°C until RT-PCR testing.

### Histological analyse of non-decalcified bone tissue

Part c of the necrotic femoral head sample (Figure. 2C) was fixed with 4% paraformaldehyde, dehydrated conventionally, cleared with xylene, and embedded in poly(methyl methacrylate) (PMMA) (Sigma-Aldrich, St. Louis, MO, USA). Then, the femoral head was cut along the coronal plane into 100-µm-thick slices with a hard-tissue-slicing machine. The slices were ground with 400-, 600-, 800-, and 1200-grit sandpaper in turn. After polishing the surface with aluminum powder (0.05 µm), and staining with hematoxylin and eosin (HE), Toluidine Blue (TB),the structural changes were observed.

### Histological analyse of decalcified bone tissue

Part *a* was subdivided into subchondral bone, necrotic, sclerotic, and healthy regions (Figure. 2C). After being fixed with 4% paraformaldehyde, decalcified with 10% ethylenediaminetetraacetic acid(EDTA) [7], dehydrated conventionally, and embedded in paraffin wax, the specimens were cut into 5-µm-thick pathological sections, stained with HE, TB, and observed.

### Immunohistochemistry

The decalcified bone tissues were embedded in paraffin and cut into 5-µm-thick sections,deparaffinized, and rehydrated.The slices were washed three times with phosphate buffered saline (PBS) and endogenous peroxidase was quenched following incubation with 3% hydrogen peroxide. Next, the samples were incubated with primary antibodies(dilution 1:50,Abcam, UK) overnight at 4°C, to detect bone morphogenetic protein 2 (BMP-2), runt-related transcription factor 2 (RUNX2), receptor activator of the nuclear factor-κB (RANK), and receptor activator of the nuclear factor-κB ligand (RANKL). After washing three times with PBS, slices were incubated with the secondary antibody(Maxim Ltd., Fuzhou, China) for 30 min followed by 3,3′-diaminobenzidine (DAB) development. Images were obtained using a BX51 Olympus microscope equipped with a DP71 camera (Olympus, Tokyo, Japan). The light source intensity of the microscope was kept constant for all tissue samples to eliminate variation.

### Alkaline phosphatase (ALP) and TRAP staining

After conventional dewaxing, the paraffin sections of different regions of the osteonecrotic femoral head specimens were subject to ALP (86R-1KT, Sigma, Aldrich) and TRAP staining (387A-1KT, Sigma, Aldrich). Osteoblast and osteoclast numbers were quantitated in the subchondral bone, necrotic, sclerotic, and healthy regions, and expressed as numbers/mm^2^. Values were calculated from at least five nonconsecutive sections per region. Images were obtained using an Olympus BX51 microscope equipped with a DP71 camera (Olympus).

### Nanoindentation test

**Figure 3:**
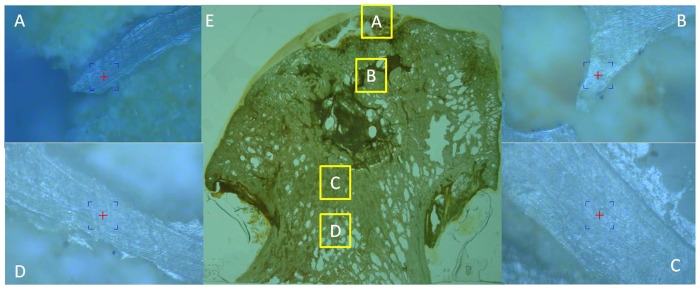
Indent positions in the nanoindentation test. Non-decalcified femoral head slices (E) of 10 randomly selected points in bone trabeculae in the subchondral bone (A), necrotic (B), sclerotic (C), and healthy(D) regions.

Specimens were prepared as described previously [8]. Samples were fixed with 4% paraformaldehyde, embedded in PMMA, cut along the coronal plane into 100-µm-thick slices, ground with 400-, 600-, 800-, and 1200-grit sandpaper in turn, and polished using a micro-cloth with 6-, 1-, and 0.05-µm alumina suspension. The non-decalcified sections were divided into four regions, as described above. The nanoindenter (MTS, USA) was equipped with a Berkovich tip. In this study, 10 indents were selected randomly in the different regions and visualized under a light microscope at 400× magnification (Figure 3). All experiments were conducted using the continuous stiffness measurement (CSM) procedure [9], and the indenter was loaded at a strain rate of 0.05 s^−1^ until reaching an indent depth of 1000 µm, after which it was held for 10 s. The allowable drift rate was kept below 1.5 nm/s for all indentations and the nanoindentations were sufficiently spaced to prevent mutual interactions.The Oliver-Pharr method was used for data analysis of the elastic modulus and hardness [10], which assumes that the tip–sample contact is purely elastic.

### Fluorescent quantitative PCR (qPCR)

Specimens from the different regions of the samples were ground into bone powder in liquid nitrogen, and then TRIzol was added to extract RNA. The concentration and purity of the RNA was evaluated by ultraviolet spectrophotometry. To a reaction volume of 20 µL, 1-µg RNA was added in a reverse transcription system, and a real-time fluorogenic quantitation PCR test was performed. The primers used (Parkson Beijing) are listed in Table 1. The reaction consisted of an initial denaturation for 30 s at 95°C, and then 40 cycles of 5 s at 95°C, 10 s at 55°C, and 15 s at 72°C, to determine the final quantification cycle (Cq) value. Using 2^−△△Cq^, gene expression was compared statistically to the osteoblast and osteoclast activity in different regions of the specimens.

**Table 1:** Primers used for quantitative real-time polymerase chain reaction.

Genes	Primer sequence
RUNX2	Forward: 5′-TGGACGAGGCAAGAGTTT- 3′
	Reverse:5′-TGGTGCAGAGTTCAGGGAG-3′
BMP2	Forward:5′-CTGTATCGCAGGCACTCA-3′
	Reverse:5′-GAATCTCCGGGTTGTTTT-3′
RANK	Forward:5′-ACGTGGACCCTTGCCCCAGT-3′
	Reverse:5′-ACTGGCCACCAGGGGAGCTT-3′
RANKL	Forward: 5′-GACATCCCATCTGGTTCCC- 3′
	Reverse:5′-AATACTTGGTGCTTCCTCC-3′
GAPDH	Forward: 5′-ATGAATGGGCAGCCGTTAG- 3′
	Reverse:5′-TGGAAGATGGTGATGGGAT-3′

### Western blotting

Western blotting was performed using proteins isolated from different regions of the 10 femoral heads. The bone tissues were washed twice with 0.9% NaCl and phosphate-buffered saline (PBS), and then lysed with NET-Triton Lysis Buffer (0.01 M Tris-Cl, 0.1 M NaCl, 1 mM EDTA pH 7.4, 1% Triton X-100, 10% glycerol, 0.1% sodium dodecyl sulfate (SDS), 0.5% sodium deoxycholate, and a cocktail of protease inhibitors). Aliquots of lysates were electrophoresed and then the proteins were transferred to poly(vinylidene fluoride) (PVDF) membranes (Bio-Rad). Nonspecific binding of the antibodies to the membrane was blocked by a 1-h incubation with 5% (w/v) non-fat dry milk/0.01 (v/v) Tween 20 in Tris-buffered saline (TBS). The membranes were probed with specific antibodies against RANK (Santa-Cruz), RANKL, OPG, RUNX2, BMP2, and BMP7 (all Abcam, UK). Human β-actin monoclonal antibody (Serotec, UK) was used as a protein marker for quantification of the protein bands. Signals were detected using secondary antibodies [anti-mouse IgG and anti-rabbit IgG conjugated to horseradish peroxidase (HRP) (dilution 1:5000, Abcam, UK)] and the membranes were immersed in ECL detection solution (Santa Cruz, USA). The protein bands were quantified using an Epson GT-8000 laser scanner. The ratios of the protein band intensities relative to that of β-actin were calculated for each sample.

### Statistical analysis

All data are presented as means ± standard deviation. The statistical software used was SPSS ver. 17.0. The groups were compared using single-factor variance analysis. Pairs of groups were compared using the least-significant-difference (LSD) test. Statistical significance was set at *P* < 0.05.

## Results

### Micro-CT evaluation

**Figure 4:**
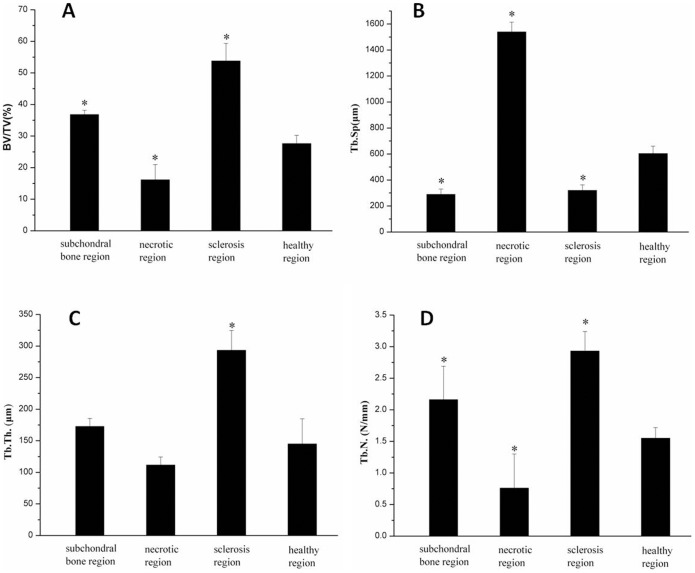
Quantitative micro-CT measurements. Measurements of (A) bone volume fraction (BV/TV), (B) trabecula space (Tb. Sp.), (C) trabecula thickness (Tb. Th.), (D) trabecula number (Tb. N.) in different regions. *P < 0.05 compared with healthy region.

In the micro-CT images (Figure 1), the arrangement of bone trabeculae and structural characteristics differed significantly in the subchondral bone, necrotic, sclerotic, and healthy regions. The bone trabeculae in the subchondral and necrotic regions were cracked and broken, but appeared thicker in the sclerotic region. The bone trabeculae appeared healthy in the normal region [11],

Lower mean values of BV/TV, Tb.Th, and Tb.N and higher mean values of Tb.Sp were observed in the necrotic region compared to those in the healthy region in samples, In addition, the microstructure of bone was changed in the sclerotic region, with increased Tb.Th and decreased Tb.Sp compared to the healthy region (Figure 4).

### Pathology evaluation

**Figure 5:**
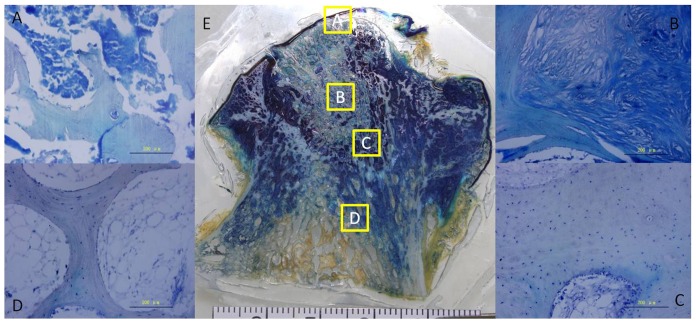
Toluidine blue staining of undecalcified and decalcified bone tissue. Toluidine blue staining of a paraffin section of the subchondral bone (A), necrotic (B), sclerotic (C), and healthy(D) regions. Toluidine blue staining of an undecalcified bone tissue slice of an entire section (E).

In the non-decalcified pathological section, the collapse and deformation of the femoral head was obvious (Figure 5). The structure of the subchondral bone had essentially disappeared and there was even separation of the joint cartilage. The area of focal necrosis was clearly demarcated from the surrounding tissue. The bone trabeculae were disorganized, and their structure and continuity lost. Bone tissue was replaced by fibrous tissue in the necrotic region. In the sclerotic region, the bone trabeculae showed bone hyperplasia, and the arrangement was compact and regular. The arrangement of bone trabeculae was normal in the healthy region, and the structure was intact.

**Figure 6:**
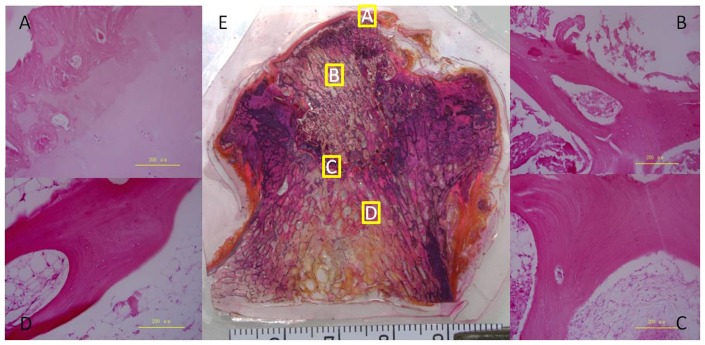
H&E staining of undecalcified and decalcified bone tissue. HE staining of a paraffin section of the subchondral bone (A), necrotic (B), sclerotic (C), and healthy(D) regions. HE staining of an undecalcified bone tissue slice of an entire section (E).

In decalcified paraffin sections, in the subchondral bone region, the bone trabeculae were narrow, and the structure had changed, with pits of empty bone and fewer bone cells (Figure 6A). In the necrotic region, the bone trabeculae were broken and bone cells were absent from the bone lacuna in the necrotic region (Figure 6B). There was degeneration and necrosis in the bone marrow cavity cell, accompanied by hyperblastosis, including connective tissue and new blood vessels. In the sclerotic region, there were large numbers of new bone trabeculae, which were tightly arranged with increased density and surrounding osteoblasts (Figure 6C). In the healthy region, the bone trabeculae were intact and the hematopoietic tissues and medullary cavity were normal (Figure 6D).

### Immunohistochemistry, TRAP, and ALP staining

**Figure 7:**
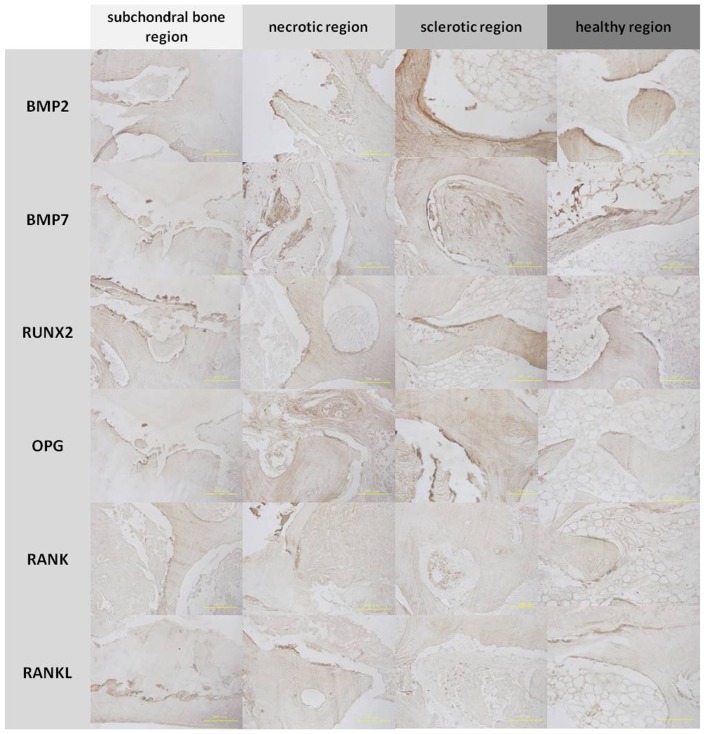
Immunohistochemistry of bone trabeculae in the regions of the femoral head. Bone morphogenetic protein 2 (BMP2), bone morphogenetic protein 7 (BMP7), Runt-related transcription factor 2 (RUNX2), and osteoprotegerin (OPG) were expressed in the bone matrix of trabeculae in the sclerotic and healthy regions. Receptor activator of nuclear factor-κB (RANK) and receptor activator of nuclear factor-κB ligand (RANKL) were expressed in the bone matrix of trabeculae in the subchondral bone and necrotic regions.

**Figure 8:**
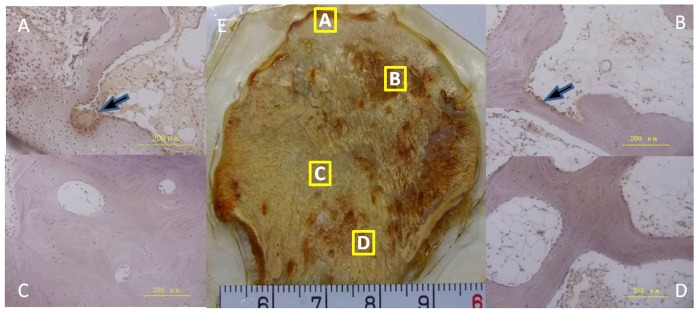
Histology of tartrate-resistant acid phosphatase (TRAP) staining. Black arrows indicated TRAP-positive cells in the subchondral bone (A) and necrotic regions (B). A reduced number of ALP-positive cells was observed in the sclerotic (C) and healthy (D) regions. TRAP staining of an undecalcified bone tissue slice of an entire section (E).

**Figure 9:**
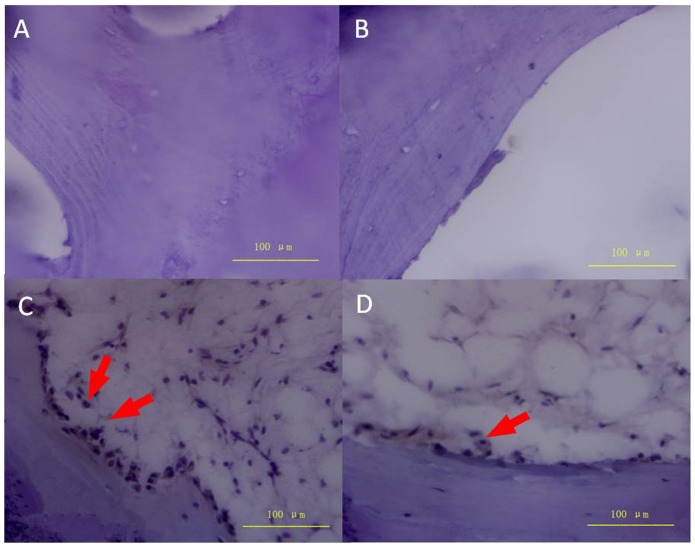
Histology of alkaline phosphatase (ALP) staining. A reduced number of ALP-positive cells were observed in the subchondral bone (A) and necrotic regions (B). Red arrows indicate ALP-positive cells in the sclerotic (C) and healthy (D) regions.

**Figure 10:**
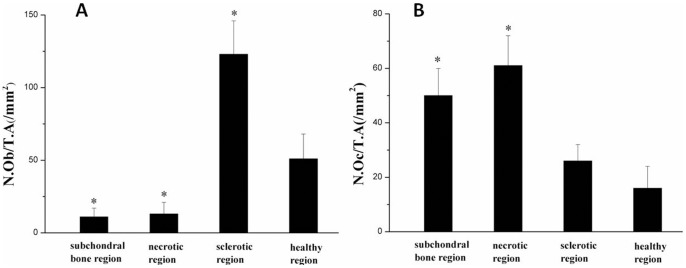
Quantitative analysis of the number of osteoblasts and osteoclasts. (A) The number of osteoblasts per unit tissue area in the subchondral bone, necrotic, sclerotic and healthy regions (N.Ob/T.A mm^2^); (B) The number of osteoclasts per unit tissue area in the subchondral bone, necrotic, sclerotic and healthy regions (N.Oc/T.A mm^2^) * *P* < 0.05 compared with the healthy region.

The subchondral bone and necrotic region were positive for RANK and RANKL, and the sclerotic region was positive for BMP2, BMP7, RUNX2, and OPG by immunohistochemical staining(Figure 7). Osteoblast (ALP-positive) cells were detected around the trabeculae in the sclerotic region, with a decreased cell number in the subchondral bone and necrotic region compared to the healthy region (Figure 8). Osteoclast (TRAP-positive) cells were detected around the trabeculae in the subchondral bone and necrotic region, with a decreased cell number in the sclerotic region compared to the healthy region (Figure 9). The mean number of osteoblast (ALP-positive) cells was increased in the sclerotic region, but decreased in the subchondral bone and necrotic region compared to the healthy region (*P* < 0.05; Figure 10A).However, the mean number of osteoclast (TRAP-positive) cells was increased in the subchondral bone and necrotic region, but decreased in the sclerotic region compared to the healthy region (*P* <0.05; Figure 10B).

### Nanoindentation test

**Figure 11:**
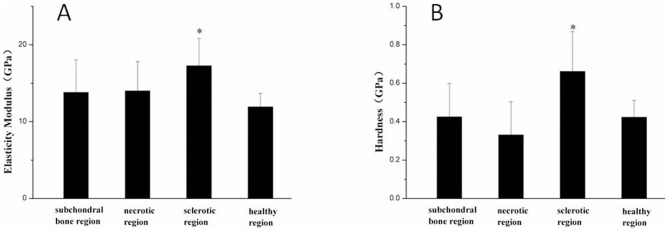
The elastic modulus and hardness of bone trabeculae in various regions. The elastic modulus (A) and hardness (B) in the various regions of the femoral head. *P < 0.05 compared with the healthy region.

The elastic modulus and hardness were significantly (*P* < 0.05) higher in the sclerotic region than the necrotic and healthy regions. However, no significant (*P* > 0.05) differences in the elastic modulus and hardness were detected between the necrotic and healthy regions (Figure 11).

### qPCR and Western blot quantitative analyses

**Figure 12:**
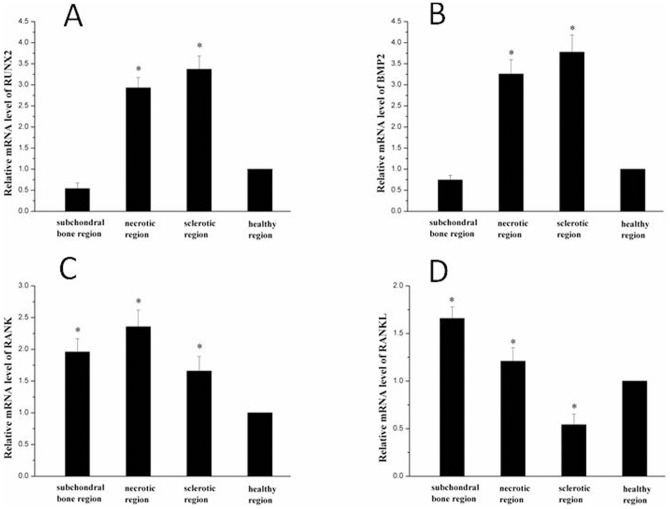
PCR results. The expression of RUNX2 (A) and BMP2 (C) was greatest in the sclerotic region (*P*< 0.05). The expression of RANKL (B) was greatest in the subchondral bone region (*P* < 0.05) and the expression of RANK was greatest in the necrotic region (D) (*P* < 0.05). *P < 0.05 compared with healthy region.

The resultd of qPCR showed that the expression of RUNX2 and BMP2 was greatest in the sclerotic region (*P* < 0.05; Figure 12 A,B). The expression of RANK and RANKL was greatest in the necrotic region and subchondral bone (*P* < 0.05; Figure 12 C,D)

**Figure 13:**
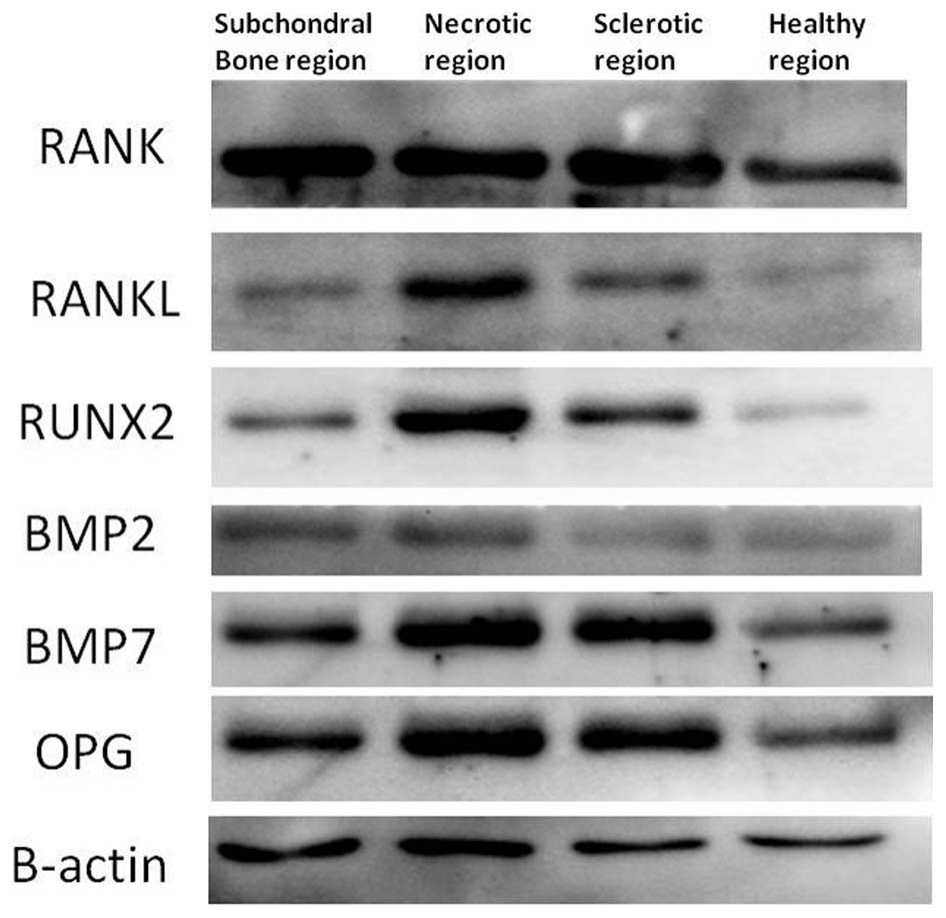
Western blot results. The expressions of RANK, RANKL, OPG, RUNX2, BMP2, and BMP7 were greater in the subchondral bone (A),necrotic (B) and sclerotic(C) regions than healthy(D) region, and highest in the necrotic region (B).

**Figure 14:**
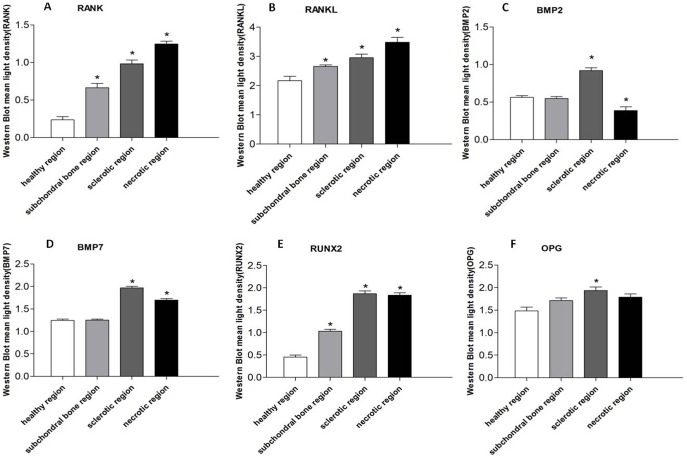
Quantitative results of Western blot analysis for the expression of RANK, RANKL, BMP2, BMP7, RUNX2, and OPG. The expressions of RANK (A) and RANKL (B) were higher in the sclerotic region compared to the healthy region, and highest in the necrotic region (*P* < 0.05). The expressions of BMP2 (C), BMP7 (D), RUNX2 (E), and OPG (F) were higher in the necrotic region compared to the healthy region and highest in the sclerotic region (*P* < 0.05). *P < 0.05 compared to the healthy region.

The expressions of RANK, RANKL, BMP2, BMP7, RUNX2, and OPG were visualized by Western blotting (Figure 13). According to the quantitative results, the expression of RANK and RANKL were higher in the sclerotic region compare to healthy region, and highest in the necrotic region (P < 0.05; Figure 14 A,B). The expressions of BMP2, BMP7, RUNX2, and OPG were higher in the necrotic region compare to the healthy region and highest in the sclerotic region (P < 0.05; Figure 14 C,D,E,F).

## Discussion

Osteonecrosis of the femoral head is a complex disease in which circulatory disorders suppress bone cell metabolism irreversibly. The sequestrum is formed after osteonecrosis. The basic pathological change is the disappearance of osteoblasts from the sequestrum surface; they are replaced by osteoclasts surrounding the sequestrum, which dissolve it to form a bone defect, resulting in the disruption of bone trabeculae and femoral head osteonecrosis and collapse.

This study used nanoindentation to determine the micromechanical properties of femoral head osteonecrosis specimens in different regions. There was no obvious difference in the elastic modulus and hardness of the bone trabeculae in the necrotic and healthy regions. Hengsberge *et al*. [12]found that the micromechanical properties of bone tissue do not directly affect the overall macromechanical properties of bone tissue. Therefore, collapse of the osteonecrotic femoral head is unlikely to be caused by reduced micromechanical properties of bone trabeculae in the weight-bearing region. In addition, the micro-CT evaluation and bone histomorphometry analysis showed that BV/TV was significantly decreased in the necrotic region,which directly determined the cancellous bone compressive strength. Increased bone turnover and porosity offset some of the positive effects on bone strength; exceeding the value of this strength in the femoral head region causes a femoral head collapse [13], [14].

Bone reconstruction requires precise coordination of bone resorption and bone formation. Osteoblasts are derived from bone marrow mesenchymal stem cells, and are responsible for the synthesis, secretion, and mineralization of bone matrix. Osteoclasts are derived from the mononuclear precursor cells of hematopoietic stem cells, and are large multinucleated cells. In the bone marrow microenvironment, osteoclast precursor cells differentiate into mature osteoclasts and regulate bone resorption. The number and function of osteoblasts and osteoclasts are regulated by a variety of factors and signaling pathways, so that bone formation and resorption remain in balance [15], [16]. Consequently, we believe that the change in the activity of osteoblasts and osteoclasts plays an important role in the course of osteonecrosis of the femoral head.

Many studies [17], [18] have shown that the OPG/RANKL/RANK system plays an important role in adjusting the balance between osteoblast and osteoclast activities, preventing bone loss and ensuring the renewal of normal bone. Many hormones and cytokines regulate osteoclast differentiation and maturation by altering OPG and RANKL synthesis by stromal cells. Generally, when the ratio of RANKL/OPG increases, the number and activity of osteoclasts increase. The normal reconstruction of bone and the stability of the bone mass depend on the balance of OPG and RANKL. RUNX2 is a specific transcription factor of osteogenesis differentiation. It can increase the transcription of genes encoding various mineralization-related proteins in osteoblasts and cartilage cells, and prompt these cells to differentiate in the direction of osteoblasts. RUNX2 also plays an important role in bone growth; it regulates the differentiation and activity of osteoblasts and osteoclasts by interacting with a variety of cytokines associated with bone metabolism [19], [20]. BMP2 and BMP7 also have strong bone-inducing activity [21]–[23].

In this study, we found TRAP-positive cells in the subchondral and necrotic regions of the osteonecrotic femoral head. These regions were also positive for RANK and RANKL by immunohistochemical staining. RANK and RANKL play an important role in regulating the process of lytic lesion formation and bone restoration [24]. RANKL is secreted by osteoblasts and binds to RANK on the surface of osteoclasts. In a study of the interaction between osteoclasts and osteoblasts. To et al [25] revealed that osteoclasts are highly dynamic, extend and retract large protrusions, and migrate towards osteoblasts. In addition, cell death was observed frequently in RANKL-induced cells that failed to establish osteoblast contact and in those that were in tight connection with osteoblasts. The distance between osteoclasts and osteoblasts is important for the survival of osteoclasts. Moreover, both osteoclasts and osteoblasts have a significant effect on each other regarding differentiation and function.The sclerotic region was positive for BMP2 and RUNX2 on immunohistochemical staining. We believe that osteoclast activity is enhanced in the subchondral and necrotic regions and osteoblast activity is enhanced in the sclerotic region of the osteonecrotic femoral head. In addition, RT-PCR showed that the expression of RUNX2 and BMP2 decreased in the subchondral region while RANK and RANKL expression increased significantly. This indicates that osteoclast activity is high and sclerotin is destroyed via non-absorption, leading to the appearance of microfractures in the subchondral bone. In addition, the expression levels of RUNX2, BMP2, and RANK are increased in the necrotic and sclerotic regions, reflecting increased osteoclast activity, which might be related to the collapse mechanism in femoral head necrosis.

We believe that the damage to the bone structure and changes in macromechanical properties lead to collapse during the process of bone restoration. The destructive bone repair process is an inherent phenomenon, in which bone destruction precedes restoration and repeated action leads to fatigue fractures. After bone restoration commences, neovascularization occurs, and new bone formation is required to break the sequestrum. The continued mechanical load results in collapse of the femoral head. If there is no bone resorption, no osteogenesis can occur. In the repair process, both osteoblasts and osteoclasts are involved. Osteogenesis requires ∼3 months to establish new bone with good mechanical performance, while the osteoclastic process needs only ∼3 weeks to affect the bone structure of the trabeculae. Therefore, the macromechanical strength of the femoral head as a whole is reduced during the repair process and the femoral head collapses under mechanical load. Therefore, we postulate that if the changes leading to femoral head necrosis and collapse can be delayed by alteration of the balance between osteoblast and osteoclast activities and provision of sufficient mechanical support, healing might be possible.

Many drugs and factors regulate the functions of osteoblasts and osteoclasts, such as bisphosphonate drugs, which inhibit osteoclast function [26]–[28], and Nell-1 factor, which promotes the differentiation of bone marrow stromal cells into osteoblasts [29], [30]. We believe a treatment that regulates osteogenesis and osteoclasts, while conferring adequate mechanical support to the femoral head, is the future of femoral head osteonecrosis therapy.
